# Case Report: Pathological Complete Response in a Lung Metastasis of Phyllodes Tumor Patient Following Treatment Containing Peptide Neoantigen Nano-Vaccine

**DOI:** 10.3389/fonc.2022.800484

**Published:** 2022-02-08

**Authors:** Huizi Sha, Qin Liu, Li Xie, Jie Shao, Lixia Yu, Lanqi Cen, Lin Li, Fangcen Liu, Hanqing Qian, Jia Wei, Baorui Liu

**Affiliations:** ^1^ The Comprehensive Cancer Centre of Nanjing Drum Tower Hospital, The Affiliated Hospital of Nanjing University Medical School, Nanjing, China; ^2^ Department of Pathology of Nanjing Drum Tower Hospital, The Affiliated Hospital of Nanjing University Medical School, Nanjing, China

**Keywords:** personalized, neoantigen, nano-vaccine, phyllodes tumor, combination therapy

## Abstract

Some of the mutant peptides produced by gene mutation transcription and translation have the ability to induce specific T cells, which are called new antigens. Neoantigen-based peptide, DNA, RNA, and dendritic cell vaccines have been used in the clinic. In this paper, we describe a lung metastasis of a phyllodes tumor patient demonstrating pathological complete response following treatment containing personalized multi-epitope peptide neoantigen nano-vaccine. Based on whole-exome sequencing (WES), RNA sequencing, and new antigen prediction, several mutated peptide fragments were predicted to bind to the patient’s human leukocyte antigen (HLA) allotypes, including ten peptides with high predicted binding affinity for six genes. The pulmonary metastases remained stable after the four cycles of anti-PD1 and anlotinib. After the addition of the multi-epitope peptide neoantigen nano-vaccine, the tumor began to collapse and contracture developed, accompanied by a decrease of tumor markers to normal, and complete pathological remission was achieved. With the use of the vaccination, recombinant human granulocyte-macrophage colony-stimulating factor (rhGM-CSF) was used every time, and low-dose cyclophosphamide was injected every 3 weeks to improve efficacy. Peripheral blood immune monitoring demonstrated immune reactivity against a series of peptides, with the most robust post-vaccine T-cell response detected against the HLA-DRB1*0901-restricted SLC44A5 V54F peptide.

## Introduction

Phyllodes tumor of the breast is a kind of tumor with hyperplasia of stroma and epithelium of breast, and phyllodes structure often appears. According to the state of hyperplasia of stroma, it is generally divided into benign, borderline, and malignant. In either case, phyllodes have the potential for local recurrence. Fields et al. reported a retrospective analysis of patients with primary breast sarcoma with a postoperative recurrence rate of 53.8% (7/13) (1). The core principle of local therapy for phyllodes tumors, whether benign or malignant, is local excision to negative margins to achieve definitive local control. However, phyllodes tumors in their most aggressive form can recur with distant metastases, histologically degenerating into a sarcomatous lesion lacking an epithelial component. The current National Comprehensive Cancer Network (NCCN) guidelines suggest that metastatic patients should be managed according to the clinical practice guidelines for soft tissue sarcomas (2).

Systemic treatment of soft tissue sarcoma mainly includes chemotherapy and molecular-targeted drug therapy, but the overall effect is not ideal. In addition, the commonly used chemotherapy drugs for soft tissue sarcoma, such as adriamycin and ifosfamide, have serious side effects. For tissue sarcoma, there are few clinical studies on checkpoint inhibitors, including SARC028 and Alliance A091401 (3–5). Arotinib can inhibit tumor angiogenesis and induce tumor cell apoptosis by targeting multiple signal pathways and can be used in the second-line treatment of soft tissue sarcoma.

The tumor vaccine is an important part of tumor immunotherapy. In recent years, with the development of various biomedical technologies, tumor vaccine research has also made a breakthrough. With the advent of the intelligent era of high-throughput gene sequencing and big data analysis, neoantigen individualized vaccines have emerged in immunotherapy (6–8). The neoantigen is produced by the tumor. Immune checkpoint-based therapies provide non-specific immune activation, but the antitumor immune responses are mediated largely through the activation of T lymphocytes recognizing mutated peptides presented by human leukocyte antigen (HLA) molecules at the tumor cell surface (9–11).

Although it has been found that tumors are polygenic disorders related to diseases for a long time in the past, only recently through high-throughput gene sequencing analysis can we clearly realize that there are dozens or even more gene mutations in almost every solid tumor. The same type of tumor occurs in different patients, and the mutation spectrum is also different. Even in the same patient, the gene mutation spectrum of the metastatic tumor is different from that of the local tumor after treatment. Some of the mutant peptides produced by gene mutation transcription and translation have the ability to induce specific T cells, which are called new antigens. In this way, teams from the United States and Germany have prepared new antigen vaccines for melanoma and glioma, which can significantly delay tumor recurrence. After the recurrence of individual patients, PD-1 antibodies can be combined to completely relieve the disease (6, 7). In this paper, we describe a lung metastasis of an Asian phyllodes tumor patient demonstrating pathological complete response (pCR) following personalized multi-epitope peptide neoantigen nano-vaccine.

## Case Report

We present the case of a 57-year-old woman. On January 2, 2014, left breast mass resection was done, and the size of the mass is 5 cm * 4 cm * 4 cm. During the operation, intraoperative rapid pathology indicates phyllodes tumors (medium grade). The patient was not followed up regularly after the first operation until 1 year later, and she found breast mass again. On January 7, 2015, left breast mass resection was performed, and the postoperative pathology showed the following: breast phyllodes tumor, 2 masses measuring 3.2 cm * 3 cm * 2.8 cm and 1.8 cm * 1.5 cm * 1.5 cm. The tumor mesenchymal cells are moderately heteromorphic, mitotic images are more common (>5/10 hpf), and some areas have infiltrative growth and tend to be borderline. The tumor tissue was close to the cutting edge (<1 mm). The patient recovered well after the above two operations and did not receive radiotherapy and chemotherapy.

On October 29, 2015, she underwent left breast mass resection for the third time, and postoperative pathology exhibited fusiform cell sarcoma, with phyllodes tumor recurrence and high possibility of sarcomatosis (**Supplementary Figure 1**). Subsequently, she underwent left mastectomy + left axillary lymph node dissection under general anesthesia on November 11, 2015. Postoperative pathology showed that a fusiform tumor was found in the local area of the residual cavity. After the operation, 6 cycles of epirubicin combined with liposomal paclitaxel chemotherapy was carried out since December 31, 2015. On July 7, 2016, radiotherapy was done to the left chest wall and the upper and lower left clavicle area. The radiotherapy dose was 2 Gy * 25 f, and the process was smooth. After regular reexamination, there was no obvious sign of recurrence and metastasis.

**Figure 1:**
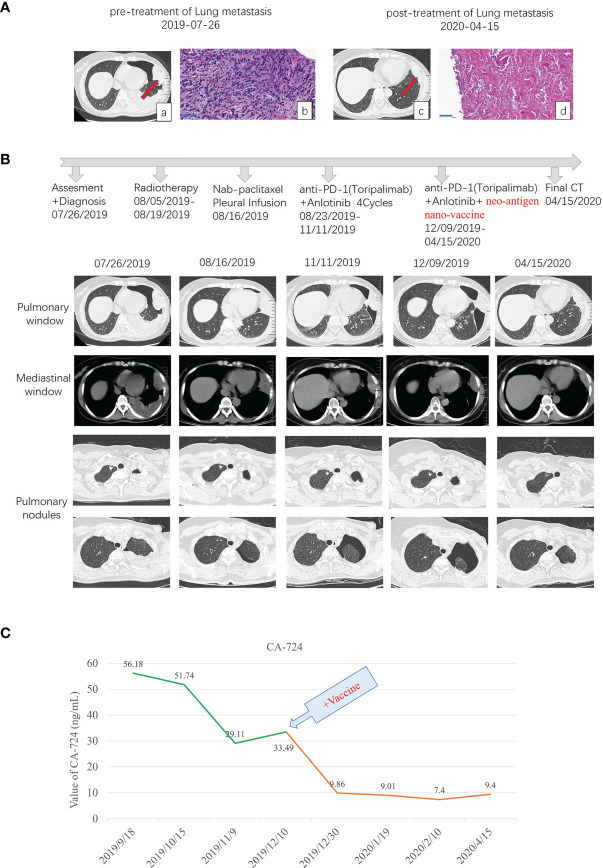
Diagnostic assessment, treatment, and clinical monitoring of lung metastasis of phyllodes tumor patient. **(A)** Pretreatment and posttreatment assessment of lung metastasis, as determined by chest CT scans **(a, c)** and pathological examination of H&E-stained needle biopsies **(b, d)**. Bar = 50 μm. **(B)** Timeline indicating treatment dates of radiotherapy, intraperitoneal chemotherapy of albumin paclitaxel, anti-PD1 combined with anlotinib treatment, and the addition of the multi-epitope peptide neoantigen nano-vaccine. The corresponding chronological series of the pulmonary window and mediastinal window CT scans are shown. Some other small lung nodules gradually vacuolated (shown in B pulmonary nodules). **(C)** The dynamic changes of the tumor marker CA-724 with time are shown.

In May 2019, CT examination showed that the density shadow of soft tissue in the left lower lobe of the lung and some other small lung nodules, and the tumor marker CA72-4 was significantly increased without anticancer treatment. In July 2019, CT reexamination showed that the density shadow of soft tissue in the left lower lobe of the lung increased compared with the previous one. On July 26, 2019, under the guidance of B-ultrasound, a percutaneous biopsy of the biggest metastasis mass was performed (**Figures 1A-a**), and next-generation genetic sequencing was performed on the tissue. The pathological results showed that there was diffuse growth of spindle cells with obvious interstitial mucus degeneration (**Figure 1A-b**). *Immunohistochemistry*: tumor cells express PD-L1 (sp142) (tumor cells −, interstitial lymphocytes −), PD-1 (lymphocytes −), HER2 (0), estrogen receptor (ER) (−), Ki67 (about 10% +), and progesterone receptor (PR) (−). The comprehensive evaluation showed the progress of the disease.

From August 5 to 19, spiral tomography of left lung metastasis mass was performed. A total of PGTV 50G/10f and PTV 30Gy/10f were completed. During the period, the patient was treated with intraperitoneal chemotherapy of albumin paclitaxel on August 16, and no obvious adverse reactions were observed. Toripalimab combined with anlotinib was performed for 4 cycles from August 23 to November 11, 2019, during which the review of the efficacy evaluation showed stable disease (SD; Response Evaluation Criteria in Solid Tumors (RECIST) 1.1). On September 18, the tumor marker CA-724 was significantly lower than before (July 25), which was due to radiotherapy (306.6 ng/ml on July 25 to 56.18 ng/ml on September 18). Examination imaging on September 19 and November 11 showed that the lesions in the left upper chest wall were mildly smaller than before (efficacy evaluation also showed SD) (images on September 19 were not shown in this paper), combined with CA-724 slightly lower than before (56.18 ng/ml on September 18 to 29.11 ng/ml on November 11).

As the metastatic lesion changed slowly and tumor marker CA-724 remained stable (**Figure 1C**), she has been given additional personalized multi-epitope peptide neoantigen nano-vaccine starting on December 9, 2019. The neoantigen nano-vaccine was designed based on the unique somatic mutation profile present in her tumor, which was detected with next-generation genetic sequencing. The patient expressed a strong desire to receive the vaccine and was given consent accordingly. The patient underwent whole-exome sequencing (WES), RNA sequencing, and new antigen prediction, which were carried out in the OrigiMed company. Her tumor mutational burden (TMB) is 0.7 Muts/MB (percentile < 80%, TMB-L), and microsatellite instability (MSI) is microsatellite stable (MSS) (**Supplementary Table 1**). Twenty-two non-synonymous somatic mutations were detected off the core tumor gene list by WES, PTEN-FAS fusion was detected by RNA sequencing (**Supplementary Table 2**), and mutation containing peptide fragments encoded by the mutated genes were assessed for predicted binding to the patient’s HLA class I and class II molecules using peptide binding prediction algorithms (12, 13). Several mutated peptide fragments were predicted to bind to the patient’s HLA allotypes, including ten peptides with high predicted binding affinity for six genes (**Table 1**).

**Table 1:** Personalized neo-epitope vaccine peptides and predicted HLA binding.

No.	Gene	Mutant amino acid	HLA type	No. of peptide	Sequence of neoantigen	Mutant peptide affinity (nM)	VAF (%)
1	CSF1R	p.L91Wfs*21	C*03:04	9	EAAPPSTSM	47	18
2	GPRC5D	p.G344V	A*02:01	10	KLSPQQDAGV	291	25
3	PHLPP1	p.I1079L	A*02:01	9	KLKAIPTTL	463	24
4	PHLPP1	p.I1079L	DRB1:09*01	15	LKAIPTTLMNCRRMH	493	24
5	SLC44A5	p.V54F	DRB1:09*01	15	FLGLVAWVHGDPRRA	455	22
6	VILL	p.R252L	A*02:01	9	QLQKANVLL	360	17
7	VILL	p.R252L	DRB1:09*01	15	PSKDINQLQKANVLL	392	17
8	PTEN-FAS	Fusion	C*03:04	9	VARLSSKSV	163	–
9	PTEN-FAS	Fusion	DRB1:09*01	15	KKVLTSVARLSSKSV	113.78	–
10	PTEN-FAS	Fusion	DRB1:09*01	15	RDKKVLTSVARLSSK	150.44	–

HLA, human leukocyte antigen; VAF, variant allele frequency.

**Figure 2:**
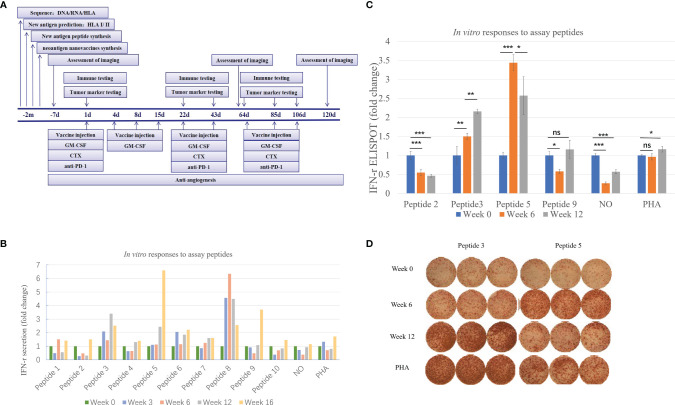
Immune monitoring of vaccination-induced peripheral blood T-cell response. Peripheral blood mononuclear cells (PBMCs) collected prior to vaccination (Week 0) and at Weeks 6 and 12 post-vaccination were assessed for peptide-specific T-cell recognition using three assays (some tests were conducted at Week 3 and Week 16). **(A)** Flowchart of vaccine treatment. **(B)** Summary of IFN-γ ELISA results showing the change in peptide-specific IFN-γ secretion by pre- and post-vaccine patient PBMCs following culture with vaccine or control. See **Table 1** for peptide designation codes and full peptide sequences. **(C)** Summary of IFN-γ ELISpot results showing the change in peptide-specific IFN-γ-producing PBMCs compared with pre-vaccine levels. **(D)** IFN-γ ELISpot results for the two mutant peptides compared with PHA controls. Statistical analyses were performed using Fisher’s least significant difference. *p < 0.05, **p < 0.01, ***p < 0.01. ns, not significant.

The injection time points of the vaccine were Day 1, Day 4, Day 8, Day 15, Day 22, Day 43, Day 64, Day 85, and Day 106 (**Figure 2A**). Recombinant human granulocyte-macrophage colony-stimulating factor (rhGM-CSF) was used with the vaccine every time, and low-dose cyclophosphamide (CTX) was injected every 3 weeks to improve efficacy. GM-CSF has been shown to stimulate monocytes and neutrophils, reducing the risk for febrile neutropenia in patients with cancer. In this case, GM-CSF was used to enhance cellular immunity based on the performance augmenting the numbers and activity of dendritic cells (DCs) (14). CTX is a systemic chemotherapeutic agent, which can eradicate immune cells, including inhibitory Tregs. Many clinical trials used CTX to regulate immunity (15–18).

After 6 injections of the vaccine, the biggest lung metastasis was gently shrunk, and after 9 injections, this tumor collapsed further. The preparation before the vaccine, the injection time of 9 vaccines, and the time point of immune detection are all shown in **Figure 2A**. This lung lesion originally showed a fusiform shape and then gradually collapsed into the pleura (**Figures 1A-c**, **1B**). After the biopsy, it was confirmed that the pathology was completely relieved (**Figures 1A-d**). Some other small lung nodules gradually vacuolated (**Figure 1B**). The course of treatment was smooth with rash and flu-like symptoms. After that, she received maintenance therapy with PD-1 antibody and anlotinib until the disease progressed and new lesions appeared, and the duration of response (DOR) is 105 days.

## Methods and Results

### Ethics and Patient Consent

The personalized multi-epitope peptide neoantigen nano-vaccine protocol for this patient was approved by the ethics committee of Nanjing Drum Tower Hospital, Nanjing, China. Informed consent was obtained from the patient and her family.

### Generation of Personalized Multi-Epitope Peptide Neoantigen Nano-Vaccine

Based on next-generation sequencing (NGS) and bioinformatics, somatic mutations were identified by analysis of tumor tissue and matching peripheral blood samples using whole-exome sequencing (WES). The mutation containing peptide fragments encoded by the mutated genes was assessed for predicted binding to the patient’s HLA class I and class II molecules using peptide binding prediction algorithms. Individual HLA-binding epitopes were selected as target peptides for the vaccine design according to the predicted major histocompatibility complex (MHC)-I H-2Kk binding affinity, and multiple neoantigen peptides were conjugated to amphiphilic lipids 1,2-distearoyl-sn-glycero-3-phosphoethanolamine-*N*-[hydroxysuccinimidyl(polyethyleneglycol)] (DSPE-PEG2000-NHS) to generate personalized multi-epitope peptide neoantigen nano-vaccine. Montanide ISA-51 was applied to form a depot at the local injection site to delay the release of antigen, and it has the effects of pro-inflammatory and recruiting antigen-presenting cells and increasing the number of lymphocytes in the lymph node drainage area. In many species, ISA-51 can induce good humoral and cellular immune responses (19). The detailed steps of preparing the vaccine will be reported in our other publications later.

### Assessment of Vaccine-Induced Antigen-Specific Cytotoxic T-Cell Response

To confirm the immunogenicity of candidate neoantigens for this patient at a series of time points pre- and post-vaccination, peripheral blood was collected from the patient prior to immunization (Week 0) and again at Weeks 3, 6, 12, and 16 post-vaccination. Screening *in vitro* IFN-γ assays on peripheral blood mononuclear cells (PBMCs) stimulated with synthesized neoantigen peptides were performed to detect and monitor antigen peptide-specific cytotoxic T lymphocyte (CTL). Please refer to the **Supplementary Materials** for specific methods.

As shown in **Figure 2B**, the secretion level of IFN-γ represents the patient’s response to each peptide. This patient received a total of ten peptide-containing nano-vaccines nine times.

Judging from the secretion level of IFN-γ in the supernatant, the patient’s response to each peptide after each cycle of injection showed a dynamic change. IFN-γ secretion levels were significantly increased (>3-fold) against 4 of the 10 mutated peptides compared to pre-vaccine levels and increased (>2-fold) against 5 of the 10 mutated peptides compared to pre-vaccine levels (**Figure 2B**). Four peptides were selected for ELISpot detection. The results of ELISpot showed that the immune response of the patient’s antigen-specific T lymphocytes to these four peptides exhibited dynamic changes over time (**Figures 2C, D**). Through a comprehensive evaluation of the results of supernatant IFN-γ secretion level and ELISpot, it can be seen that the selected four peptides are sorted according to immunogenicity in the order of peptide 5>3>9>2. This also corroborated that the supernatant IFN-γ secretion level can to a certain extent, instead of the results of ELISpot, reflect the patient’s response to each peptide. In summary, immune monitoring demonstrated immune reactivity against a series of peptides, with the most robust post-vaccine T-cell response detected against the HLA-DRB1*0901-restricted SLC44A5 V54F peptide.

### Cytometric Analysis of T Lymphocyte and Cytometric Bead Array Analysis of Cytokines

Flow cytometry was used to quantify the activation of T cells after vaccinations, and patients’ peripheral T cells were collected and labeled with several antibodies for cytometry analysis of the proportions of different types of T cells. Please refer to the **Supplementary Materials** for specific methods.

With the injection of the vaccine, the distribution of lymphocyte subsets changed in different degrees (**Table 2**). After 6 weeks of vaccination, the proportion of CD3+ T cells did not change much, but the proportion of CD3+CD8+ T cells increased slightly. The changes of CD3+CD8+CD45RO+CD62L− effector memory T cells and CD3+CD8+CD45RO+CD62L+ central memory T cells were opposite. At the 6th week, the proportion of CD3+CD8+CD45RO+CD62L+ central memory T cells increased, while the proportion of CD3+CD8+CD45RO+CD62L− effector memory T cells decreased. The percentage of CD11c + cells increased in the 6th week. Cytokine IFN-γ and TNF-α were also increased after 6 weeks of vaccination.

**Table 2:** The distribution of lymphocyte subsets and the secretion of cytokine.

No.	Antibody	Results (%)	
		Week 0	Week 6
1	CD3+	59.87	60.45
2	CD3+CD4+	41.95	40.09
3	CD3+CD8+	18.86	21.18
4	CD3+CD8+CD45RO+CD62L+	10.14	19.86
5	CD3+CD8+CD45RO+CD62L−	54.05	40.33
6	CD3+CD4+CD45RO+CD62L+	35.10	21.98
7	CD11C+	12.28	15.72
**No.**	**Cytokine**	**Week 0**	**Week 6**
1	IFN-γ	27.21	57.79
2	TNF-α	18.50	20.00

## Discussion

Phyllodes tumors are fibroepithelial breast tumors capable of a diverse range of biological behavior. Surgical margins are the best predictor of phyllodes tumor local recurrence, with wide margins (typically >1 cm and potentially >2 cm) associated with the lowest risk of recurrence. For this patient, the tumor tissue was close to the cutting edge (<1 mm) for the operation on January 7, 2015. After the third operation, spindle cell sarcoma was considered in the pathological diagnosis of the patient. Phyllodes tumor was more likely to relapse into sarcomas. Therefore, extended surgery and postoperative adjuvant radiotherapy and chemotherapy were performed.

Three years later (in 2019), lung metastases occurred, and lung metastases were confirmed by biopsy as malignant phyllodes tumor of the breast. Moreover, the molecular type of lung metastases was triple negative, PD-L1 (sp142) (tumor cell −, interstitial lymphocyte −) and PD-1 (lymphocyte −). After gene sequencing, the patient’s TMB was also very low, which was 0.7 Muts/MB, and belonged to the MSS type. After radiotherapy of the biggest metastasis mass, the tumor marker CA-724 had a rapid decline. Subsequently, even if PD-1 antibody and anlotinib were used in combination, the change of CA-724 was still stable. It was not until the addition of personalized multi-epitope peptide neoantigen nano-vaccine that CA-724 was reduced to nearly normal. We guess that the tumor microenvironment changes from cold tumor to hot tumor, so it can benefit from comprehensive immunotherapy and achieve pCR.

*Lancet Oncology* published the data of a phase II clinical trial, in which PD-1 antibody combined with axitinib was used to treat soft tissue sarcoma, especially for alveolar soft part sarcoma, which was recognized as the most sensitive subtype to immunotherapy (20). Therefore, the guidelines recommend that PD-1 inhibitors can be combined with antiangiogenic drugs in soft tissue sarcomas. In this case, even if PD-1 antibody and anlotinib were used in combination, the imaging assessment is still SD, and the change of CA-724 was stable. After the personalized multi-epitope peptide neoantigen nano-vaccine was added, the good effect appeared. In a phase Ib clinical trial, Ott et al. demonstrated the feasibility, safety, and immunogenicity of the combination of personalized neoantigen vaccines and PD-1 inhibition in patients with advanced solid tumors (melanoma, non-small cell lung cancer, and bladder cancer) (21). To our knowledge, this is the first case of soft tissue sarcoma in tumor vaccine therapy that achieved clinical response.

In recent decades, clinical trials of tumor vaccines singlely used for solid tumors have mostly failed, especially in the case of heavy tumor load. Combined immunotherapy including PD-1 antibody, anti-angiogenesis, and even radiotherapy will bring new hope to patients (22). If we reexamine radiotherapy from the perspective of the anti-tumor immune effect, it is not difficult to find that the immune system is closely involved in the whole process of radiotherapy. First of all, radiotherapy can induce tumor cell apoptosis and necrosis and release tumor antigen (especially new antigen) into the blood to promote immune recognition. Secondly, radiotherapy can mediate tumor cells to release dangerous signals, such as high mobility group box 1 (HMGB1) and adenosine triphosphate, so that DCs can recognize the damaged tumor cells and phagocytize them; HMGB1 can also induce DCs to differentiate and phagocytize the damaged tumor cells. When the DCs mature, they present antigen to T cells more effectively; activated DCs migrate to regional draining lymph nodes, present tumor cell-specific antigen to naive T cells, and promote their activation into effector T cells. Finally, multiple chemokines produced by radiotherapy also help effector T cells return to tumor tissue to exert an immune effect. In addition, through the distant effect, radiotherapy can even play a similar immune effect on tumors outside the target area. Therefore, Taichiro Goto, a Japanese scholar, thinks that radiotherapy can be regarded as the production of tumor *in situ* vaccine and autonomous vaccination process (23).

Nanotechnology was initially known for the enhanced permeability and retention (EPR) effect, which was used as passive targeting loading cytotoxic drugs. In the field of immunotherapy, nanotechnology is gradually emerging. In vaccine technology, the introduction of nanotechnology has the following advantages: 1) nanoparticles are easily concentrated in the lymph nodes, spleen, and other lymphatic organs (the size of nanoparticles is generally selected at 5–100 nm); 2) it can load many different drugs at the same time and play a role in the same time and space; and 3) drug slow release and controlled release function can avoid antigen degradation and prolong the half-life of loaded antigen. Compared with the traditional naked peptide subcutaneous immunization, the number of new antigen reactive T cells can be increased by 30 times after the antigen peptide and adjuvant are loaded with nanoparticles, and the tumor growth is effectively inhibited (24). However, the preparation process of nano-vaccines is time-consuming, and the screening method of new antigenic peptides needs more support.

This patient achieved pCR after comprehensive treatment including the vaccine, which may indicate that vaccines may have a broader prospect in the early stage of the disease, with low tumor load and even in the adjuvant treatment stage.

## Ethics Statement

The studies involving human participants were reviewed and approved by the Ethics Committee of Nanjing Drum Tower Hospital. The patients/participants provided their written informed consent to participate in this study. Written informed consent was obtained from the individual(s) for the publication of any potentially identifiable images or data included in this article.

## Author Contributions

HS is responsible for managing the patient, preparing the vaccine, and writing the paper. QL is responsible for designing the neoantigen peptide and vaccine. LX is responsible for the patients’ PD-1 antibody therapy, anlotinib therapy, and biopsy. JS is responsible for the ELISpot assay. LY is responsible for cytokine detection. LC and HQ are responsible for preparing the vaccine. LL and FL are responsible for pathological sections and consultation. BL and JW are responsible for revising the paper and formulating the patient treatment plan. All authors listed have made a substantial, direct, and intellectual contribution to the work and approved it for publication.

## Conflict of Interest

The authors declare that the research was conducted in the absence of any commercial or financial relationships that could be construed as a potential conflict of interest.

## Publisher’s Note

All claims expressed in this article are solely those of the authors and do not necessarily represent those of their affiliated organizations, or those of the publisher, the editors and the reviewers. Any product that may be evaluated in this article, or claim that may be made by its manufacturer, is not guaranteed or endorsed by the publisher.
