# Supplementary material for: Case Report: Pathological Complete Response in a Lung Metastasis of Phyllodes Tumor Patient Following Treatment Containing Peptide Neoantigen Nano-Vaccine
Source: Front Oncol. 2022 Feb 8;12:800484. doi: 10.3389/fonc.2022.800484 (PMC8861377; doi:10.3389/fonc.2022.800484)
Supplement: Supplementary file 2 [file Table_1.docx]

Supplementary material **Table1.** Gene Sequence Information of the patient.

| Gene Sequence Information | |
| --- | --- |
| Gender | Female |
| Age | 57y |
| MSI | MSS |
| TMB | 0.7 Muts/Mb |
| PD-L1 (sp142) | tumor cells – and interstitial lymphocytes - |
