# Supplementary material for: Case Report: Pathological Complete Response in a Lung Metastasis of Phyllodes Tumor Patient Following Treatment Containing Peptide Neoantigen Nano-Vaccine
Source: Front Oncol. 2022 Feb 8;12:800484. doi: 10.3389/fonc.2022.800484 (PMC8861377; doi:10.3389/fonc.2022.800484)
Supplement: Supplementary file 3 [file Table_2.docx]

Supplementary material **Table2.** Genetic sequencing analysis of patient lung tumor.

| Gene | Transcript_Ref | C_dot | P_dot | Freq |
| --- | --- | --- | --- | --- |
| CCNL2 | NM_001039577 | c.481G>A | p.V161M | 0.17 |
| C1orf168 | NM_001004303 | c.1442C>G | p.S481C | 0.18 |
| SLC44A5 | NM_001130058 | c.160G>T | p.V54F | 0.22 |
| ATP1A4 | NM_144699 | c.88dup | p.M30Nfs*22 | 0.27 |
| GPRC5D | NM_018654 | c.1031G>T | p.G344V | 0.25 |
| ZNF768 | NM_024671 | c.751C>T | p.R251W | 0.20 |
| PHLPP1 | NM_194449 | c.3235A>C | p.I1079L | 0.24 |
| FBXO15 | NM_001142958 | c.416T>A | p.M139K | 0.21 |
| FKRP | NM_001039885 | c.758C>T | p.A253V | 0.23 |
| ZNF638 | NM_001014972 | c.2500+10T>C |  | 0.18 |
| STRADB | NM_001206864 | c.93+9del |  | 0.10 |
| VILL | NM_015873 | c.755G>T | p.R252L | 0.17 |
| SCN5A | NM_000335 | c.3949G>C | p.E1317Q | 0.16 |
| FYCO1 | NM_024513 | c.3277A>G | p.K1093E | 0.20 |
| GC | NM_000583 | c.607-10T>A |  | 0.25 |
| BMP2K | NM_017593 | c.536G>A | p.R179Q | 0.21 |
| MMAA | NM_172250 | c.444G>C | p.L148F | 0.18 |
| PRDM9 | NM_001310214 | c.2347C>T | p.R783W | 0.16 |
| CSF1R | NM_005211 | c.271del | p.L91Wfs*21 | 0.18 |
| PDGFRB | NM_002609 | c.1684T>G | p.Y562D | 0.24 |
| PRPS1L1 | NM_175886 | c.25G>A | p.G9S | 0.17 |
| DGKI | NM_001321708 | c.2943G>T | p.M981I | 0.20 |
| PTEN-FAS | NM_000314; NM_152872 | - | PTEN exon5-FAS exon2 fusion | -* |

Twenty-two nonsynonymous somatic mutations were detected of the core tumor gene list by WES. * PTEN-FAS fusion was detected by RNA seq.
